# Bioinformatics and Gene Expression Omnibus Analysis of Key Candidate Genes and Pathways Associated with Femoral Head Necrosis

**DOI:** 10.5812/ijpr-145223

**Published:** 2024-06-02

**Authors:** Yuanjing Ding, Yuxia Ma, Heng Yan, Enshui Zhang

**Affiliations:** 1 Department of Joint Surgery, Central Hospital Affiliated to Shandong First Medical University, Shandong, China; 2 Department of Rheumatology and Immunology, Central Hospital Affiliated to Shandong First Medical University, Shandong, China; 3 Department of Anesthesiology, The Second Hospital, Cheeloo College of Medicine, Shandong University, Shandong, China; 4 Department of Joint Surgery, Jinan Central Hospital, Cheeloo College of Medicine, Shandong University, Shandong, China

**Keywords:** Femoral Head Necrosis, Differentially Expressed Genes, Gene Expression Omnibus, STITCH Database, Docking Study, DFT Analysis

## Abstract

**Background:**

Femoral head necrosis (FHN) is a debilitating bone disease affecting an estimated 8 million people worldwide. Although specific drugs for FHN have limitations, targeted therapies have shown promising results. The significance of this study is underscored by the high prevalence of FHN, the limitations of current treatments, and the potential of targeted drugs and natural compounds for effective therapeutic interventions.

**Objectives:**

This study aimed to explore the genetic landscape and associated pathways of FHN through bioinformatics analysis of Gene Expression Omnibus (GEO) data and molecular docking simulations targeting specific enzymes implicated in FHN.

**Methods:**

Differentially expressed genes (DEGs) in FHN samples were identified from GEO datasets, specifically accession number GSE123568 (Platform: GPL15207). Functional enrichment analysis was performed using the Database for Annotation, Visualization, and Integrated Discovery (DAVID) to identify enriched pathways and Gene Ontology (GO) terms. Additionally, a protein-protein interaction (PPI) network was constructed using the STITCH (search tool for interaction of chemicals) database, which helped identify top hub genes and proteins. Molecular docking was conducted against key proteins using compounds from the topical chinese herbal medicine (TCHM) database associated with FHN.

**Results:**

The study provided a comprehensive bioinformatics analysis of key candidate genes and pathways associated with FHN, which may serve as potential therapeutic targets. It was found that FHN is associated with mitogen-activated protein kinases (MAP4K4/ MAPK8/ MAPK9) and interleukins (IL1b/ IL19/ IL26). Molecular docking results showed strong interactions of traditional Chinese herbal compounds through hydrogen bonding and electrostatic interactions at the active sites of the top ten target proteins associated with FHN.

**Conclusions:**

The study confirmed that FHN is linked with enzymes such as mitogen-activated protein kinases (MAPKs), interleukins, tumor necrosis factors (TNFs), and VEGFA (vascular endothelial growth factor A). Molecular docking simulations demonstrated that hesperidin, naringin, and curcumin exhibit potent inhibition against key proteins involved in FHN. Future research will focus on elucidating the specific roles of genes associated with FHN and exploring potential therapeutic targets using natural compounds.

## 1. Background

Femoral head necrosis (FHN) is a prevalent, debilitating bone disease primarily affecting the middle-aged and elderly population (1). It is estimated that around 8 million people over 20 years old in China suffer from FHN (2). The disease not only has a high morbidity rate but also a notably low cure rate, posing a significant threat to patients and potentially causing severe economic burdens for their families (3). The disease progresses to the collapse of the articular cartilage of the femoral head, leading to early onset osteoarthritis (4). Although surgical interventions can alleviate symptoms, the complex pathogenic mechanisms of FHN are not fully understood, complicating treatment and early prevention (5). The pathology of FHN is thought to be multifaceted, associated with factors like abnormal lipid metabolism, ischemia, and apoptosis (5). It is recognized that impaired blood perfusion contributes to FHN by hindering normal cellular repair functions and causing irreversible tissue damage (6). Timely and accurate diagnosis, coupled with conservative therapeutic approaches, might be more effective than surgical methods for managing the condition (7). Thus, understanding the specific pathogenesis of FHN and identifying precise early diagnosis and treatment techniques is critical (8). Although the use of specific drugs for FHN has limitations, targeted therapies have shown potential in treating this complex disease (9). Furthermore, microarray data analysis has become a valuable tool for investigating various diseases, including FHN, by identifying potential biomarkers and elucidating molecular mechanisms through gene profiling (10). Advances in bioinformatics tools and techniques have significantly enhanced gene microarray data analysis, offering new insights for the diagnosis and treatment of many diseases at the genetic level (11). Femoral head necrosis predominantly affects individuals aged 30 to 50 and is more common in men than women, with a male-to-female ratio of approximately 8:1. The exact global prevalence of FHN is not well documented; however, it is estimated to affect around 20,000 to 30,000 individuals annually in the United States alone. Risk factors for FHN include trauma, corticosteroid use, alcohol consumption, and certain medical conditions such as sickle cell disease and systemic lupus erythematosus. Without appropriate treatment, FHN can lead to severe pain, joint dysfunction, and ultimately necessitate joint replacement surgery. Early diagnosis and intervention are crucial to prevent further damage and preserve joint function. The novelty of this study lies in its interdisciplinary approach, which involves identifying specific genes, pathways, and therapeutic targets, exploring natural compounds, assessing molecular interactions, and considering personalized medicine in the treatment and prevention of FHN. These elements collectively enhance the research's uniqueness and importance. By using a variety of methods and tools, the study delves into the pathophysiology of FHN and seeks potential therapeutic targets.

## 2. Objectives

This research aimed to identify key genes and pathways associated with FHN through bioinformatics analysis, target specific enzymes related to FHN via molecular docking, and examine the potential of natural compounds as inhibitors of critical proteins linked to FHN. This comprehensive approach seeks to significantly advance the understanding of FHN and the development of effective treatment strategies.

## 3. Methods

### 3.1. Data Resource

**Figure 1.**
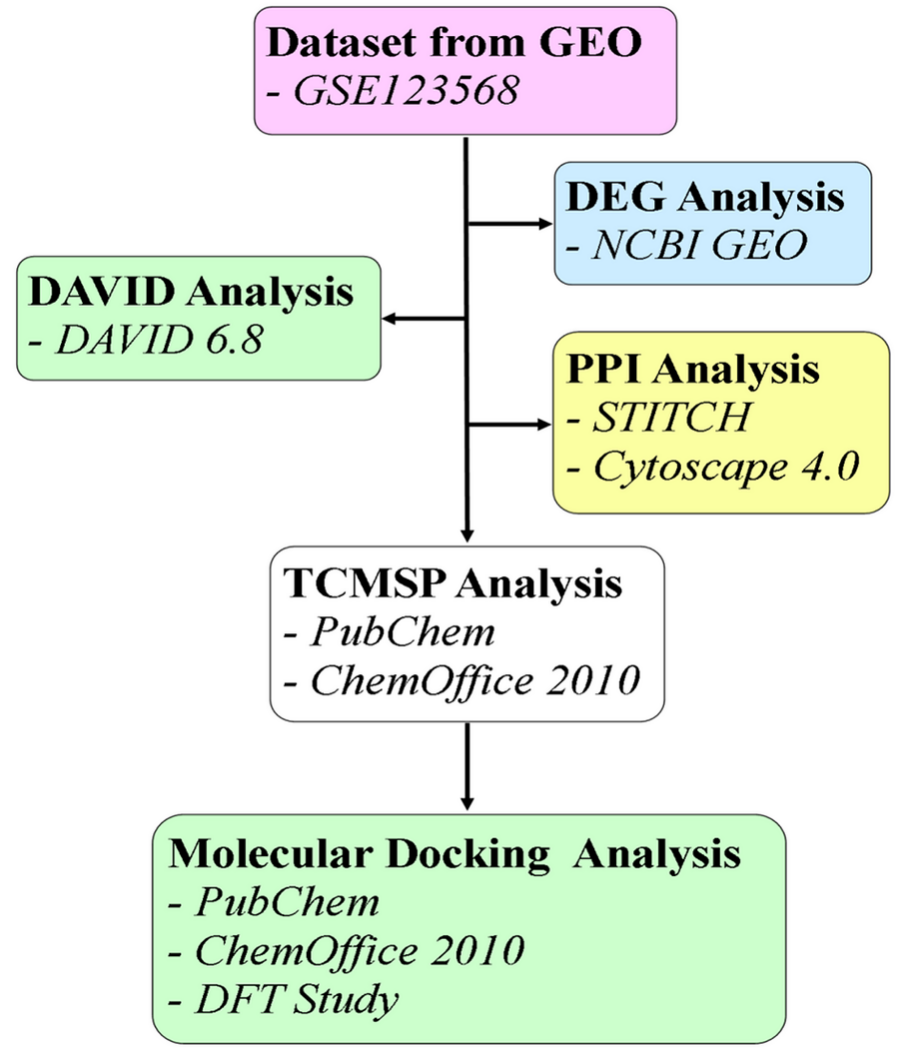
A Flowchart illustrating the workflow of the present study.

The Gene Expression Omnibus (GEO) is a vital resource in molecular biology and genetics for analyzing gene expression patterns across different conditions. Gene Expression Omnibus allows researchers to compare and contrast gene expression data from various experiments and tissues, facilitating the detection of biomarkers, understanding of disease mechanisms, and identification of potential drug targets. The expression microarray datasets related to FHN (Homo sapiens) were retrieved from the NCBI repository, GEO, using the accession number GSE123568 (Platform: GPL15207) (12). This dataset includes a total of 40 samples, with 30 from the disease group and 10 controls. Figure 1 presents a flowchart illustrating the structured pipeline for the methodology employed in this study.

### 3.2. Data Preprocessing and Differentially Expressed Gene (DEG) Analysis

The retrieved differentially expressed gene (DEG) microarray data was analyzed using the NCBI GEO database. Data preprocessing was conducted using a software package, and quality control checks were performed to ensure the reliability of the processed data. Statistical methods were employed to identify DEGs. The chosen thresholds for the statistical results were applied to identify genes that are significantly differentially expressed between groups. Differentially expressed genes were determined based on a significant cutoff, incorporating considerations such as false discovery rate control, power analysis, and biological significance.

### 3.3. Database for Annotation, Visualization, and Integrated Discovery (DAVID) Analysis

The Database for Annotation, Visualization, and Integrated Discovery (DAVID) was utilized for analysis using version 6.8. Database for Annotation, Visualization, and Integrated Discovery is a widely-used bioinformatics resource for the functional annotation and enrichment analysis of genes and proteins. The biological pathways involved in FHN were analyzed. Pathway enrichment analysis in DAVID 6.8 helped identify biological pathways significantly enriched with genes or proteins of interest. After interpreting the pathway enrichment, statistical significance was determined based on a significant cutoff and other considerations such as false discovery rate control, power analysis, and biological relevance.

### 3.4. Protein-Protein Interaction (PPI) Analysis

The PPI network of the DEGs associated with FHN was mapped using the STITCH database, accessible via the STITCH (Search Tool for Interactions of Chemicals) website (https://stitch.embl.de/). Additionally, Cytoscape 4.0 was used to construct the core targets of FHN using the STITCH database. This analysis may provide functional annotations of the proteins associated with the progression of FHN and help in targeting specific proteins or enzymes by inhibiting their function.

### 3.5. TCMSP Analysis

A search was conducted in the Traditional Chinese Medicine Systems Pharmacology Database and Analysis Platform (TCMSP, http://tcmspw.com/index.php) to retrieve a set of ligands and compounds associated with FHN. The details of the compounds retrieved from the TCMSP database are provided in appendix 1 of the supplementary information. These compounds were optimized using MM2 force fields in ChemOffice 2010 (Perkin Elmer, USA) and converted to Sybyl mol2 format.

### 3.6. Molecular Docking Simulation

Molecular docking was carried out using the MolexusMolegro Virtual Docker (MVD 7.0, Molexus, Denmark). The cavity detection feature of MVD was used to identify potential ligand binding sites. This step aims to predict the binding affinity between identified compounds and target proteins related to FHN, thereby identifying potential drug candidates that may interact effectively with these proteins. The 3D structures of target proteins, such as Cyclin-dependent kinase (PDB ID: 1BI7), estrogen receptor (PDB ID: 1HCQ), IL-1beta (PDB ID: 9ILB), mitogen-activated protein kinase (PDB ID: 1DI9), cyclooxygenase-2 (PDB ID: 6BL3), Tnfrsf1b (PDB ID: 3ALQ), and vascular endothelial growth factor A (PDB ID: 6ZCD), were obtained from the Protein Data Bank (http://www.rcsb.org/). Water molecules associated with these protein structures were removed as they do not contribute to the docking scoring function. Furthermore, the binding sites of these proteins were predicted and established at the following coordinates: X: 147.14, Y: 44.94, Z: 86.62 (volume: 51.71 A3, surface: 186.88 A2) for PDB ID: 1BI7; X: 27.10, Y: 6.77, Z: 94.93 (volume: 11.88 A3, surface: 67.68 A2) for PDB ID: 1HCQ; X: -15.49, Y: 5.50, Z: 1.87 (volume: 23.55 A3, surface: 84.48 A2) for PDB ID: 9ILB; X: 42.61, Y: 24.02, Z: 31.58 (volume: 403.45 A3, surface: 1176.32 A2) for PDB ID: 1DI9; X: -45.78, Y: -17.74, Z: 32.74 (volume: 188.42 A3, surface: 506.88 A2) for PDB ID: 6BL3; X: 11.56, Y: 16.29, Z: -49.25 (volume: 12.28 A3, surface: 51.2 A2) for PDB ID: 3ALQ; and X: 37.07, Y: 36.59, Z: -5.39 (volume: 30.21 A3, surface: 121.6 A2) for PDB ID: 6ZCD. The flexibility of bonds and amino acid side chains was adjusted within the restriction sphere of the cavity. The RMSD (root mean squared deviation) threshold was set at 2.00 Å with an energy penalty of 100.00. A minimum of 30 docking engine runs were performed, and the best orientation from 50 runs was selected for detailed analysis.

### 3.7. DFT Studies

DFT studies were conducted based on the MM2 optimized geometry of the docked compounds exported using MVD. These DFT calculations, performed using the DFT-B3LYP/6–31G basis set, help in understanding the molecular mechanics of the docked compounds. This analysis aims to elucidate the intermolecular interactions that may contribute to the interaction with the protein. The comprehensive methodology employed in this study uses various methods and tools to thoroughly investigate the molecular mechanisms and potential therapeutic interventions for FHN. Each method was selected for its specific utility in addressing different aspects of the research objectives, ranging from gene expression analysis to protein interactions and molecular docking studies.

## 4. Results

### 4.1. Identification of Microarray Data

In this study, a total of 795 elements from 40 human samples within GSE123568 (Platform: GPL15207) were analyzed. Out of these, 195 unique elements were identified as differentially expressed in association with FHN.

### 4.2. Database for Annotation, Visualization, and Integrated Discovery (DAVID) Analysis

Database for Annotation, Visualization, and Integrated Discovery analysis helped identify the biological characteristics of the 195 unique elements. Genes positively and negatively associated with FHN were determined based on their enrichment scores and are detailed in Tables 1 and 2, respectively. The genes positively correlated with the onset of FHN included ER membrane proteins (ES = 6.38), mitochondrion inner membrane proteins (5.85), ribonucleoproteins (5.62), chaperones (4.83), and keratin-associated proteins (4.36) (Table 1). Conversely, genes negatively correlated with FHN included GPCR, metal-binding proteins, pleckstrin, leucine-rich repeat proteins, and SH3 domain proteins, all with an enrichment score of zero (Table 2). Genes directly associated with FHN include the proteasome, NIK/NF-kappaB signaling, tumor necrosis factor-mediated signaling, interleukin-1-mediated signaling, estrogen signaling, vascular smooth muscle contraction, VEGF signaling, MAPK signaling, sphingolipid signaling, and transcription factors with sequence-specific DNA binding (Table 3). The diseases most frequently associated with FHN are shown in Table 4, with Type 2 diabetes having the highest association (ES = 1.34), followed by Alzheimer's disease (ES = 1.6), AIDS (ES = 1.7), prostate cancer (ES = 1.7), and ovarian cancer (ES = 2.3).

**Table 1.** Positively Associated Top 5 Hits Linked to Femoral Head Necrosis (FHN)

Identifiers	Molecular Function	Count	Fold Change	Benjamini	Enrichment Score (ES)	P-Value
**ER membrane protein**	-	72	1.7E0	2.5E-4	6.38	5.6E-6
**Mitochondrion inner membrane protein**	Oxidative phosporyation	30	2.2E0	2.1E-3	5.85	1.6E-4
**Ribonucleoprotein**	Cytoplasmic translation	39	3.3E0	1.4E-8	5.62	1.5E-10
**Chaperone**	Protein folding	26	2.9E0	9.6E-5	4.83	3.0E-6
**Keratin associated protein**	Intermediate filament	8	1.4E1	5.4E-4	4.36	3.9E-7

**Table 2.** Negatively Associated Top 5 Hits Linked to Femoral Head Necrosis (FHN)

Identifiers	Function	Count	Fold Change	Benjamini	Enrichment Score (ES)	P-Value
**GPCR**	Transducer	10	3.0E-1	1.0E0	0	1.0E0
**Metal binding protein**	-	85	8.0E-1	1.0E0	0	9.9E-1
**Pleckstrin**	-	9	5.0E-1	1.0E0	0	1.0E0
**Leucine-rich repeatProtein **	-	3	2.8E-1	1.0E0	0	1.0E0
**SH3 domain Protein**	-	4	5.1E-1	1.0E0	0	9.9E-1

**Table 3.** Femoral Head Necrosis Associated Top 10 Hits

Identifiers	Count	Fold Change	Benjamini	Enrichment Score (ES)	P-Value
**Proteasome**	12	5.5E0	1.6E-4	2.79	9.0E-6
**NIK/NF-kappaB signaling **	8	4.4E0	2.2E-1	2.79	2.1E-3
**Tumor necrosis factor-mediated signaling pathway **	10	3.3E0	2.6E-1	2.79	3.0E-3
**Interleukin-1-mediated signaling pathway**	7	4.2E0	3.8E-1	2.79	6.1E-3
**Estrogen signaling pathway**	7	9.5E-1	1.0E0	0.44	7.6E-1
**Vascular smooth muscle contraction **	6	8.3E-1	1.0E0	0.44	8.5E-1
**VEGF signaling pathway **	5	1.6E0	1.0E0	0.44	3.9E-1
**MAPK signaling pathway **	12	7.6E-1	1.0E0	0.44	9.2E-1
**Sphingolipid signaling pathway**	7	1.1E0	1.0E0	0.44	6.2E-1
**Transcription factor activity, sequence-specific DNA binding **	10	4.5E-1	1.0E0	0	1.0E0

**Table 4.** Disease Most Frequently Associated with Femoral Head Necrosis ^a^

Diseases	Count	Enrichment Score	Benjamani	P-Value
**Type 2 diabetes**	119 (15.0)	1.34	4.2E-1	7.5E-4
**Alzheimer's disease**	64 (8.1)	1.6	6.4E-2	8.5E-5
**Acquired immunodeficiency syndrome**	63 (7.9)	1.7	4.8E-2	4.3E-5
**Prostate cancer**	36 (4.5)	1.7	5.8E-1	1.8E-3
**Ovarian cancer**	35 (4.4)	2.3	1.8E-2	8.0E-6

^a^ Values are expressed as No. (%).

### 4.3. Protein-Protein Interaction (PPI) Network Construction

**Figure 2.**
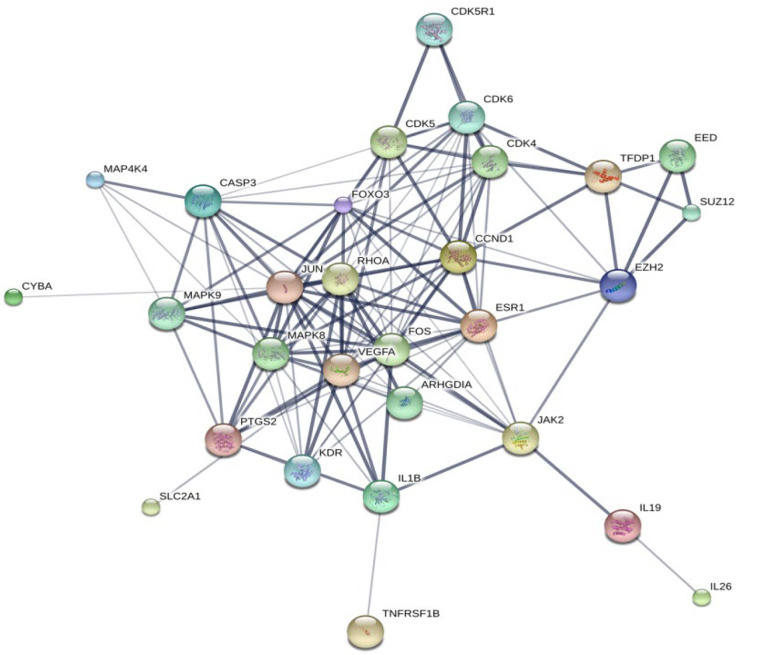
Search tool for interaction of chemicals (STITCH) database analysis showing the target proteins linked to femoral head necrosis (FHN).

A PPI network for the DEGs associated with FHN was constructed using the STITCH database and visualized with Cytoscape software version 3.8.2. The network analysis allowed us to calculate and rank the degree values of the DEGs, identifying 29 hub genes that are potentially key players in the development of FHN, as depicted in Figure 2. Table 5 lists the top 10 target proteins identified through this analysis, indicating their significant roles and pathways involved in FHN.

**Table 5.** Top 10 Target Protein Linked to Femoral Head Necrosis (FHN) from Search Tool for Interaction of Chemicals (STITCH) Database and Their PDB IDs

Protein Name	Function	PDB ID
**Casp3**	Caspase 3, apoptosis-related cysteine peptidase	Not available
**CDK (cyclindependant kinases)**	Involved in apoptotic cell death in neuronal diseases.	1BI7 (Chain A)
**ESR1 (estrogen receptor 1)**	Regulation of eukaryotic gene expression	1HCQ (Chain A)
**Interleukins**	Playing some important roles in inflammatory responses	9ILB (Chain A)
**KDR**	Kinase insert domain receptor	Not available
**MAPK (mitogen-activated protein kinase)**	Playing a role in the response to environmental stress and cytokines such as tnf	1DI9
**PTGS2**	Prostaglandin-endoperoxide synthase 2	6COX
**TFDP1**	Transcription factor Dp-1	Not available
**TNFRSF1B**	Tumor necrosis factor receptor superfamily member 1B	3ALQ (Chain R)
**VEGF-A**	Vascular endothelial growth factor A	6ZCD

### 4.4. Molecular Docking Simulation

Table 6 displays the molecular docking simulation scores for the top docking hit compounds at the active sites of various target proteins. Lower MolDock and Rerank scores typically indicate superior binding affinity, while higher interaction scores suggest stronger interactions with the target molecule. Additionally, a higher negative HBond score signifies more favorable hydrogen bonding interactions.

In the molecular docking analysis targeting cyclin-dependent kinase (PDB ID: 1BI7), hesperidin (-385.18 kJ/mol), naringin (-350.94 kJ/mol), sophoricoside (-332.47 kJ/mol), curcumin (-325.70 kJ/mol), and sesamin (-313.50 kJ/mol) stood out with the lowest MolDock, rerank scores, and total scores among the ligands. This indicates robust binding affinity and favorable interaction with the target protein, surpassing the control inhibitors ribociclib (-263.37 kJ/mol) and palbociclib (-260.15 kJ/mol), which exhibit comparatively weaker binding affinities.

Regarding the estrogen receptor (PDB ID: 1HCQ), hesperidin (-220.06 kJ/mol), capsaicin (-200.39 kJ/mol), naringin (-196.72 kJ/mol), sesamin (-182.20 kJ/mol), and curcumin (-176.98 kJ/mol) showcased the most favorable MolDock, rerank scores, and total scores compared to the control inhibitors toremifene (-141.06 kJ/mol) and tamoxifen (-124.49 kJ/mol). Notably, the absence of hydrogen bonding interactions in the control inhibitors implies a potentially weaker binding affinity.

Examining IL-1beta (PDB ID: 9ILB), arctiin (-395.28 kJ/mol), naringin (-366.85 kJ/mol), curcumin (-355.28 kJ/mol), hesperidin (-343.76 kJ/mol), and gingerol (-324.60 kJ/mol) exhibit the highest total scores and promising molecular docking scores among the ligands, indicating potentially stronger overall binding affinities and interactions compared to the control inhibitors rutaecarpine (-246.18 kJ/mol) and dexamethasone (-154.41 kJ/mol).

Among the ligands tested against mitogen-activated protein kinase (PDB ID: 1DI9), solanine (-452.1 kJ/mol) exhibits the most negative MolDock score, rerank score, and total score, suggesting the strongest binding affinity. Following closely are epigallocatechin gallate (-378.4 kJ/mol), hesperidin (-366.9 kJ/mol), and curcumin (-366.5 kJ/mol), highlighting robust binding interactions. In contrast, the control inhibitors U0126 (-266.09 kJ/mol) and PD98059 (-213.90 kJ/mol) exhibit relatively weaker total scores.

For Cox-2 (PDB ID: 6BL3), rhamnetin (-414.61 kJ/mol), hesperidin (-411.95 kJ/mol), and arctiin (-392.82 kJ/mol) present overall favorable MolDock scores, rerank scores, and total scores, indicative of strong binding affinity. The control inhibitor celecoxib (-372.67 kJ/mol) displays a comparatively lower total score.

Concerning Tnfrsf1b (PDB ID: 3ALQ), hesperidin (-316.94 kJ/mol), curcumin (-281.32 kJ/mol), epigallocatechin gallate (-258.59 kJ/mol), arctiin (-249.06 kJ/mol), and naringin (-231.63 kJ/mol) exhibit overall favorable total scores compared to the control inhibitors cannabidiol (-181.59 kJ/mol) and catechin (-170.07 kJ/mol).

Finally, for the VEGFA protein (PDB ID: 6ZCD), curcumin (-343.65 kJ/mol), shogaol (-317.59 kJ/mol), resveratrol (-318.94 kJ/mol), kaempferol (-313.01 kJ/mol), and hesperidin (-288.68 kJ/mol) demonstrate more promising molecular docking scores and total scores among the ligands. This implies potentially stronger overall binding affinities compared to the control inhibitors sunitinib (-230.19 kJ/mol) and sorafenib (-209.96 kJ/mol). In summary, hesperidin, naringin, curcumin, arctiin, and epigallocatechin gallate show the most interaction and the most favorable among the targeted proteins. These compounds demonstrate the lowest MolDock and rerank scores, suggesting the strongest binding affinity against the targeted enzymes. Among the docked compounds, hesperidin, naringin, and curcumin exhibit the strongest binding affinity with the target molecule, as they consistently rank as the top three docking hits across more than two target proteins. The total docking scores, represented by the MolDock and Rerank Scores, along with interaction energy and hydrogen bonding, offer insights into the binding affinity of these compounds with the target protein.

**Table 6.** Top 5 Docking Hits Docked Against the Target Protein Linked to Femoral Head Necrosis (FHN)

PDB ID and Ligand	MolDock Score	Rerank Score	Interaction	HBond	Total
**1BI7 **					
Hesperidin	-101.06	-104.00	-165.58	-14.54	-385.18
Naringin	-106.51	-91.06	-143.60	-9.77	-350.94
Sophoricoside	-91.31	-89.82	-143.91	-7.43	-332.47
Curcumin	-108.07	-83.32	-127.97	-6.34	-325.70
Sesamin	-108.84	-86.55	-117.77	-0.34	-313.50
Ribociclib (control)	-79.90	-76.74	-106.73	0.00	-263.37
Palbociclib (control)	-72.92	-72.47	-110.92	-3.85	-260.15
**1HCQ **					
Hesperidin	-49.15	-54.16	-110.83	-5.91	-220.06
Capsaicin	-62.06	-52.32	-83.14	-2.87	-200.39
Naringin	-42.74	-47.99	-100.99	-5.00	-196.72
Sesamin	-62.04	-43.90	-76.25	0.00	-182.20
Curcumin	-50.58	-42.88	-82.76	-0.76	-176.98
Toremifene (control)	-46.43	-31.00	-63.63	0.00	-141.06
Tamoxifen (control)	-35.61	-28.59	-60.29	0.00	-124.49
**9ILB**					
Arctiin	-129.88	-95.81	-157.70	-11.89	-395.28
Naringin	-130.71	-90.83	-141.77	-3.54	-366.85
Curcumin	-98.90	-97.12	-153.54	-5.73	-355.28
Hesperidin	-100.34	-87.88	-145.46	-10.08	-343.76
Gingerol	-107.29	-86.07	-124.75	-6.49	-324.60
Rutaecarpine (control)	-89.44	-45.95	-106.95	-3.84	-246.18
Dexamethasone (control)	-32.67	-46.28	-72.19	-3.27	-154.41
**1DI9**					
Solanine	-137.0	-125.3	-182.3	-7.5	-452.1
Epigallocatechin gallate	-116.6	-95.5	-149.9	-16.4	-378.4
Hesperidin	-98.1	-100.7	-161.5	-6.6	-366.9
Curcumin	-119.4	-99.2	-142.4	-5.5	-366.5
Astragaloside	-99.9	-100.5	-149.6	-2.6	-352.6
U0126 (control)	-85.29	-74.45	-102.67	-3.69	-266.09
PD98059 (control)	-64.82	-61.23	-87.86	0.00	-213.90
**6BL3**					
Rhamnetin	-128.18	-114.29	-164.20	-7.95	-414.61
Hesperidin	-114.50	-111.15	-172.25	-14.06	-411.95
Arctiin	-124.20	-103.42	-161.35	-3.85	-392.82
Quercetin	-117.75	-106.39	-149.33	-7.94	-381.40
Curcumin	-124.61	-105.22	-144.90	-2.63	-377.36
Celecoxib (control)	-133.11	-93.67	-143.58	-2.31	-372.67
Valdecoxib (control)	-111.63	-92.62	-116.82	-3.20	-324.27
**3ALQ**					
Hesperidin	-80.14	-83.90	-138.19	-14.71	-316.94
Curcumin	-88.68	-73.09	-114.73	-4.83	-281.32
Epigallocatechin gallate	-81.96	-62.83	-105.80	-8.00	-258.59
Arctiin	-75.28	-56.78	-109.14	-7.85	-249.06
Naringin	-58.96	-56.35	-107.31	-9.02	-231.63
Cannabidiol (control)	-60.98	-49.34	-69.74	-1.53	-181.59
Catechin (control)	-49.91	-45.19	-69.19	-5.79	-170.07
**6ZCD**					
Curcumin	-113.23	-89.04	-140.91	-0.48	-343.65
Shogaol	-104.02	-84.32	-128.46	-0.80	-317.59
Resveratrol	-110.37	-83.34	-125.02	-0.21	-318.94
Kaempferol	-97.17	-78.11	-126.88	-10.85	-313.01
Hesperidin	-73.77	-77.50	-131.17	-6.24	-288.68
Sunitinib (control)	-73.11	-60.39	-96.69	0.00	-230.19
Sorafenib (control)	-55.01	-54.25	-99.13	-1.57	-209.96

### 4.5. Molecular Interaction Analysis

**Figure 3.**
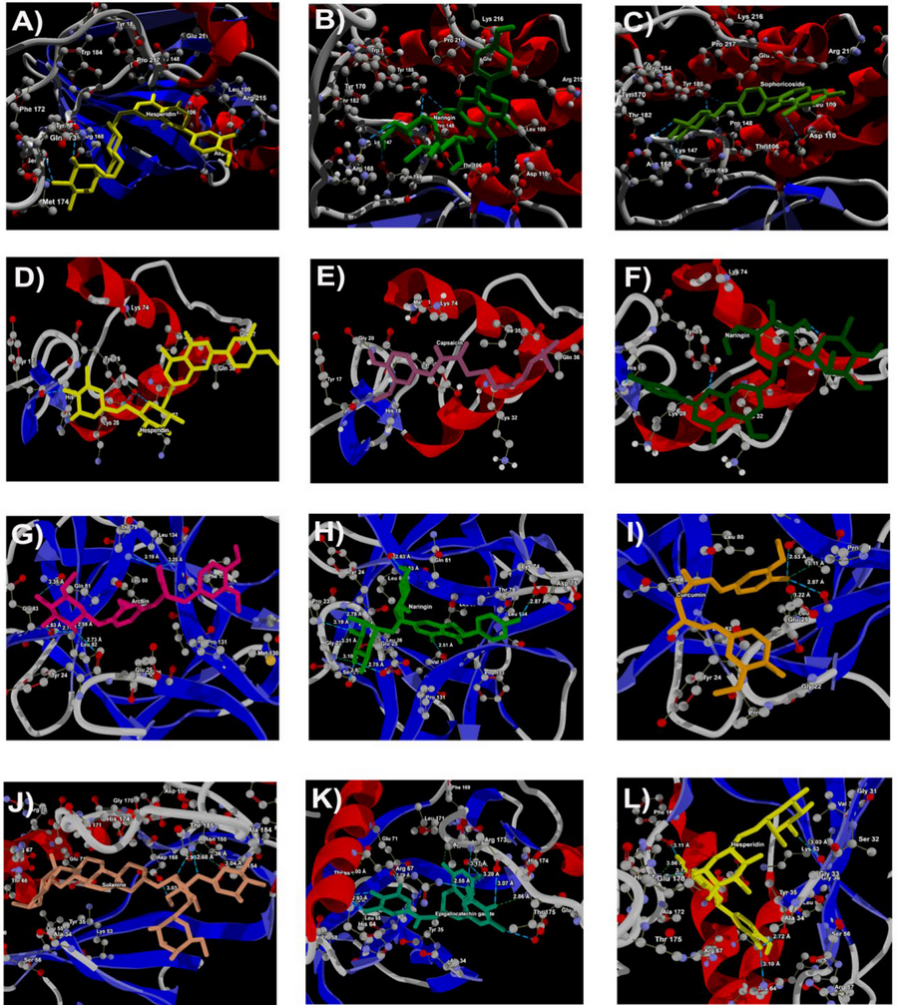
Docking of (A), hesperidin; (B), naringin; (C), sophoricosideagainst 1BI7; docking of (D), hesperidin; (E), capsaicin; (F), naringinagainst 1hcq (Esr1); docking of (G), arctiin; (H), naringin; (I), curcumin against 9ilb iL-1beta; docking of (J), solanine; (K), epigallocatechingallate; (L), hesperidin against PDB ID: 1DI9 (MAPK)

The molecular interaction analysis of the top three docking hits against the FHN-associated target proteins is detailed in appendix 2 of the supplementary information. Hesperidin demonstrated molecular interactions with residues Tyr170, Met174, Phe172, Ser171, Tyr185, Glu211, Thr106, Leu109, and Arg215 of 1BI7, spanning distances from 2.67 Å to 3.59 Å (Figure 3A). Naringin exhibited interactions with Gln149, Pro148, Tyr185, Tyr106, and Arg168 residues of 1BI7, with distances ranging from 2.70 Å to 3.29 Å (Figure 3B). Sophoricoside showed interactions with Arg168, Gln149, Tyr185, Thr106, and Asp110 residues of 1BI7, with distances ranging from 2.65 Å to 3.58 Å (Figure 3C). 

For 1HCQ, hesperidin interacted with Tyr17, His18, and Tyr19 residues, with distances ranging from 2.57 Å to 3.14 Å (Figure 3D). Capsaicin exhibited interactions with Tyr19 and Gly20, at distances of 3.00 Å and 3.25 Å, respectively (Figure 3E). Naringin displayed close interactions with His18, Tyr19, and Ile35, with distances from 2.74 Å to 2.93 Å (Figure 3F). 

In the case of 9ILB, arctiin demonstrated molecular interactions with Tyr24, Leu80, Gln81, Leu82, and Leu134 residues, spanning distances from 2.51 Å to 3.80 Å (Figure 3G). Naringin interacted with Ser21, Gly22, Pro23, Tyr24, Glu25, Lys74, Leu82, and Val132, with distances ranging from 2.51 Å to 3.80 Å (Figure 3H). Curcumin had interactions with Leu26 and Val132, with distances of 2.53 Å and 2.87 Å, respectively (Figure 3I). 

For 1DI9, solanine interacted with Lys152, Ser154, and Asp168, with distances from 3.03 Å to 3.36 Å (Figure 3J). Epigallocatechin gallate displayed interactions with His64, Arg67, Thr68, Glu71, Asp168, Ala172, Arg173, Thr175, and Glu178, with interaction distances ranging from 2.55 Å to 3.31 Å (Fig. 3K). Hesperidin also interacted with Ala34, Gly36, His64, Phe169, and Leu171, with distances from 2.72 Å to 3.13 Å (Figure 3L). 

### 4.6. Molecular Interaction Analysis

**Figure 4.**
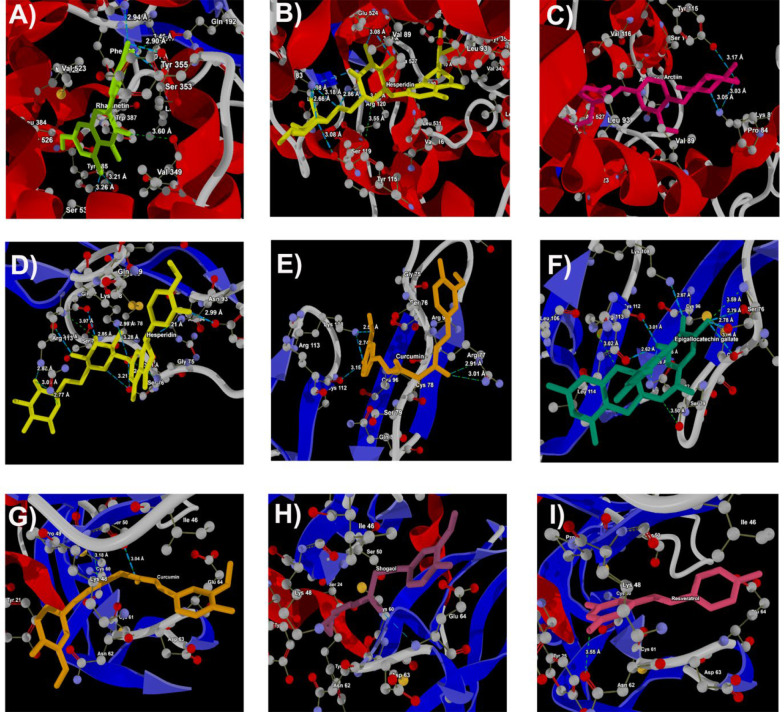
Docking of (A), rhamnetin; (B), hesperidin; (C), arctiinagainst 6BL3- Cox2; docking of (D), hesperidin; (E), curcumin; (F), epigallocatechingallateagainst 3ALQ Tnfrsf1b; docking of (G), curcumin; (H), shogaol; (I), resveratrol against6ZCD (VEGFA)

In 6BL3, rhamnetin interacted with His90, Gln192, Val349, Leu352, Ser353, Tyr385, and Ser530, with interaction distances ranging from 2.90 Å to 3.60 Å (Figure 4A). Hesperidin exhibited interactions with Lys83, Tyr115, Ser119, Arg120, Glu524, and Ser530, at distances from 2.86 Å to 3.18 Å (Figure 4B). Arctiin had interactions with Lys83 and Tyr115, at distances of 3.05 Å and 3.17 Å, respectively (Figure 4C). 

In PDB ID: 3ALQ, hesperidin demonstrated molecular interactions with Tyr61, Ser76, Ser79, Gln82, Asn93, Lys108, and Arg113, spanning distances from 2.79 Å to 3.28 Å (Figure 4D). Curcumin interacted with Arg77, Lys108, and Cys112, with distances ranging from 2.55 Å to 3.15 Å (Figure 4E). Epigallocatechin gallate showed interactions with Ser76, Arg77, Ser79, Gln82, Lys108, Cys112, and Arg113, with distances ranging from 2.67 Å to 3.06 Å (Figure 4F). 

In 6ZCD, curcumin exhibited molecular interactions with Ser50, with an interaction distance of 3.04 Å (Figure 4G). Shogaol interacted with Glu64, with an interaction distance of 3.31 Å (Figure 4H). Resveratrol demonstrated molecular interactions with Asn62, with an interaction distance of 3.55 Å (Figure 4H). 

Overall, hesperidin, naringin, and curcumin demonstrated the strongest binding affinity among the investigated compounds (Figures 3 and 4). These compounds are most likely to form stable complexes with their target proteins, exhibiting the highest interaction scores, indicating strong and favorable interactions. This could be attributed to their specific binding modes or the formation of multiple favorable interactions such as hydrogen bonds, hydrophobic interactions, or electrostatic interactions.

### 4.7. DFT Studies

**Figure 5.**
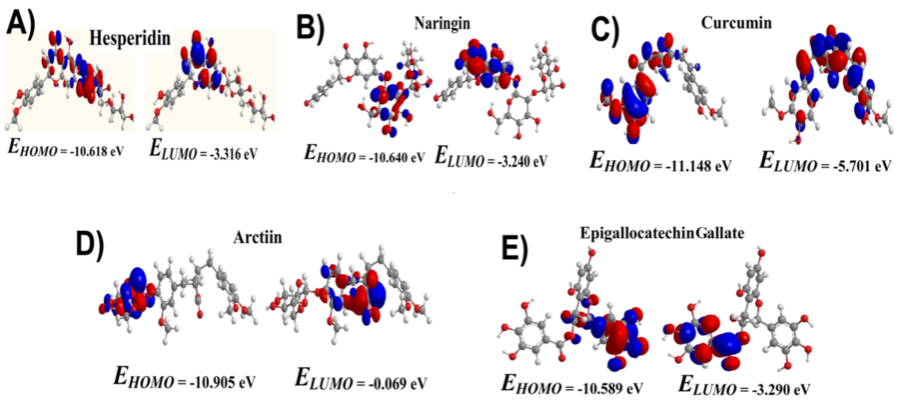
DFT analysis of depicting the HOMO and LUMO energies of (A), hesperidin; (B), naringin; (C), curcumin; (D), arctiin andepigallocatechingallate depicting the molecular orbital’s optimized at DFT/B3LYP/6-31 basis set.

DFT analysis was conducted to utilize a quantum mechanical approach for studying the electronic structure of atoms, molecules, and materials. This study focused on analyzing the band gap energy using DFT, a crucial aspect of materials science. The DFT calculations were performed using the DFT/B3LYP with a 6-31G basis set. The frontier molecular orbital energies (EHOMO and ELUMO) of the compounds were optimized at DFT-B3LYP/6-31 G basis set levels, and results are displayed in Figure 5. The band gap energy was determined from the band structure plot by finding the energy difference between the top of the valence band and the bottom of the conduction band. This energy difference represents the minimum energy required to promote an electron from the valence band to the conduction band, fundamental for understanding a material's electrical and optical properties.

## 5. Discussion

Femoral head necrosis, also known as avascular necrosis (AVN) of the femoral head, is characterized by the death of bone tissue due to disrupted blood supply (13). The pathogenesis of the disease remains unclear, and effective prevention and early treatment options are limited (14). Pathological mechanisms of FHN include abnormal lipid metabolism, microcirculation disturbances, ischemia, and apoptosis (15). Reduced blood flow is considered the primary cause, leading to impaired cellular repair functions and irreversible tissue damage (16). Previous research has often focused on bone and cartilage tissues, critical to the disease's progression.

In this study, GEO analysis identified 195 DEGs associated with FHN and oxidative stress (17). These genes are predominantly active in the cytoplasm and nucleoplasm, playing significant roles in inflammatory responses, oxidative stress, and phospholipid metabolism (18). Database for Annotation, Visualization, and Integrated Discovery analysis revealed that the target genes and proteins are mainly involved with ER membrane proteins, mitochondrion inner membrane proteins, ribonucleoproteins, chaperones, and keratin-associated proteins (19). These genes were generally up-regulated, while proteins such as GPCR, metal-binding proteins, pleckstrin, leucine-rich repeat proteins, and SH3 domain proteins showed down-regulation (20).

Enrichment analysis using DAVID and KEGG indicated that DEGs were chiefly enriched in pathways related to tumor necrosis factor-mediated signaling (including NIK/NF-kappaB and IL-1), estrogen signaling, vascular smooth muscle contraction, VEGF signaling, MAPK signaling, and sphingolipid signaling pathways. The comprehensive bioinformatics study, which included DAVID and STITCH analyses, identified 29 enriched target proteins associated with FHN (21). Among these, CASP3, CDK4/CDK5/CDK5R1/CDK6/CCND1, ESR1, IL1B/IL19/IL26, KDR, MAP4K4/MAPK8/MAPK9, PTGS2, TFDP1, TNFRSF1B, and VEGFA were highlighted as key hub proteins. Database for Annotation, Visualization, and Integrated Discovery, KEGG, and STITCH analyses recognized interleukins, PTGS2/TNFRSF1B, VEGFA, ESR1, and mitogen-activated protein kinases (MAPKs) as integral to the pathology of FHN.

Differential gene expression analysis is essential for understanding the molecular mechanisms and pathways involved in FHN. Although the precise mechanisms remain elusive, various interleukins, including IL-1β, IL-19, and IL-26, have been implicated. Elevated levels of IL-1β have been detected in femoral head tissues of patients with FHN (22), suggesting that IL-1β may contribute to the progression of FHN by inducing dysfunction in osteoblasts and osteoclasts and promoting bone resorption (23). Studies also suggest a role for IL-19 in bone remodeling and inflammation, potentially influencing osteoblast and osteoclast activity, crucial for bone homeostasis. However, it is essential to recognize that FHN's pathogenesis is multifactorial, and the involvement of interleukins is just one aspect of this complex disease process (24).

Several MAPKs, including MAP4K4, MAPK8 (JNK), and MAPK9 (p38), have been investigated for their potential association with FHN. Increased expression and activation of MAP4K4 in the femoral head tissues of affected individuals suggest its potential role in promoting osteoblast apoptosis and impairing bone remodeling processes. Inhibiting MAP4K4 activity has shown promise in preclinical studies as a therapeutic approach to prevent or treat FHN (25). Activation of MAPK8 (JNK) in FHN indicates its potential role in the disease process. Moreover, elevated p38 activity may contribute to the production of inflammatory cytokines like IL-1β and TNF-α, promoting dysfunction in osteoblasts and osteoclasts and ultimately causing bone tissue damage (26). Targeting p38 signaling has been explored as a potential therapeutic strategy for managing FHN.

However, the involvement of cyclin-dependent kinases such as CDK4, CDK5, CDK5R1, CDK6, and CCND1 in FHN is not well understood or reported, and their role in the disease remains poorly characterized (27). While the specific roles of these CDKs in FHN are not fully elucidated, it is plausible that their dysregulation may contribute to disruption in bone remodeling processes (28). The docking study identified various energy interactions, including electrostatic, hydrophobic, steric, and hydrogen bonding interactions, at the active sites of the top 10 proteins associated with FHN.

Tadesse et al. documented the molecular interaction of the compound PD0332991 with the ATP binding site of CDK6, particularly with Val101, Gln149, and Asp163 residues (29). Our findings align with this, indicating that the top three docking hits of 1BI7 interact with Thr106. The structure of CDK6 features a bilobal fold, consisting of an N-terminal lobe (residues 1 - 100) with 5 β-sheets and an α-helix, and the ATP binding site positioned at the lobal interface. The hinge forms one edge, while the activation loop spans residues 163 - 189 (30). In the case of CDK6, our results show robust interactions between the top three docking hits and residues 163 - 189, including Tyr170, Ser171, Phe172, Met174, and Tyr185 for hesperidin; and Arg168 and Tyr185 for naringin and sophoricoside, respectively.

Another study by Vigers et al. noted that the IL1 beta binding site encompasses residues Arg11, Ser13-Gln15, Met20-Gly22, Tyr24, Lys27, and Leu29-Met36, along with Gln38, Gln126-Pro131, Thr147, and Gln149 (31). In our study, naringin interacted with active site residues Ser21 and Gly22, while arctiin showed interactions with the active site residue Tyr24. Curcumin, however, did not exhibit any interaction with these active site residues. Liu et al. reported a similar molecular interaction pattern, where a compound interacted with residues Met20, Ser21, Gly23, Tyr24, Lys27, His30, Gln32, Gln34, and Gln38 of IL1beta (30).

For MAPK, docking revealed that solanine and epigallocatechin gallate showed molecular interactions with Asp168, while hesperidin demonstrated interactions with both Asp168 and Phe169, aligning with previous reports (32).

For Cox-2, the active site is located within a long hydrophobic channel that extends from the membrane-binding domain to the core of the catalytic domain, with the binding site situated in the upper half of the channel, spanning from Arg120 to near Tyr385 and Ser530 in the channel's middle (33). Our docking analysis of 6BL3 highlighted rhamnetin's interactions with COX-2 active sites Tyr385 and Ser530, and hesperidin's interactions with Arg120 and Ser530 residues. This analysis revealed complex ligand-protein interactions within FHN-associated target proteins.

Additionally, compounds such as naringin, sophoricoside, capsaicin, arctiin, solanine, epigallocatechin gallate, curcumin, shogaol, and resveratrol displayed distinct binding profiles with their respective target proteins. These interactions validate the predicted binding sites, thereby enhancing the reliability of our molecular docking predictions. using density functional theory (DFT), we analyzed the electronic ground state of molecules like hesperidin, naringin, curcumin, arctiin, and epigallocatechin gallate to understand their stability. Density functional theory calculations allow us to evaluate the band gap energy, which reveals electronic and optical properties crucial for molecular interactions. Our research emphasizes the strong binding affinity, high interaction scores, and favorable hydrogen bonding interactions of these compounds, providing insights into their molecular interactions. Generally, these compounds are considered safe and non-toxic when used in typical dietary amounts (34). Hesperidin and naringin, flavonoids found in citrus fruits, are recognized as safe and are not reported to cause significant acute toxicity or adverse effects at typical dietary intake levels. Curcumin, the active compound in turmeric, used as a spice or food ingredient, is also regarded as safe and not known to cause significant adverse effects at typical dietary intake levels (35). Arctiin, a lignan found in various plants, is considered safe when consumed as part of the diet. Lastly, epigallocatechin gallate, a catechin found in green tea, is one of the major bioactive compounds responsible for its health benefits and is considered safe when consumed as part of a normal diet (36). It is crucial to consider dosage, individual sensitivity, and compound forms. Our detailed molecular interaction analysis illuminates how different ligands bind to FHN-associated target proteins, enhancing our understanding. By scrutinizing specificity, validating predicted binding sites, and understanding ligand-receptor complexes spatially, we advance our knowledge of these interactions, which is crucial for drug discovery and development.

### 5.1. Conclusions

In conclusion, the genes and pathways identified in this study may be linked to the molecular mechanisms of FHN. The study also highlighted the association of FHN with cyclin-dependent kinases, interleukins, MAPKs, tumor necrosis factor receptor, and vascular endothelial growth factor, suggesting their potential roles in the pathogenesis of the disease. Molecular docking simulation studies revealed that hesperidin, naringin, and curcumin possess strong inhibitory effects against the top ten proteins associated with FHN. Studies have demonstrated increased expression and activation of these enzymes in the femoral head tissues of patients with FHN, indicating their significant contribution to the underlying cellular and molecular processes.

ijpr-23-1-145223-s001.pdf

## Data Availability

The datasets generated during and/or analyzed during the current study are available from the corresponding author on reasonable request.

## References

[1] Choi HR, Steinberg ME, Y. Cheng E (2015). Osteonecrosis of the femoral head: diagnosis and classification systems.. Curr Rev Musculoskelet Med..

[2] Zhang Y, Wang X, Jiang C, Hua B, Yan Z (2022). Biomechanical research of medial femoral circumflex vascularized bone-grafting in the treatment of early-to-mid osteonecrosis of the femoral head: a finite element analysis.. J Orthop Surg Res..

[3] Wang P, Wang C, Meng H, Liu G, Li H, Gao J (2022). The Role of Structural Deterioration and Biomechanical Changes of the Necrotic Lesion in Collapse Mechanism of Osteonecrosis of the Femoral Head.. Orthop Surg..

[4] Zhang X, Deng W, Ju J, Zhang S, Wang H, Geng K (2022). A Method to Visualize and Quantify the Intraosseous Arteries of the Femoral Head by Vascular Corrosion Casting.. Orthop Surg..

[5] Mont MA, Salem HS, Piuzzi NS, Goodman SB, Jones LC (2020). Nontraumatic Osteonecrosis of the Femoral Head: Where Do We Stand Today?: A 5-Year Update.. J Bone Joint Surg Am..

[6] Hu Y, Yang Q, Zhang J, Peng Y, Guang Q, Li K (2023). Methods to predict osteonecrosis of femoral head after femoral neck fracture: a systematic review of the literature.. J Orthop Surg Res..

[7] Chen Y, Miao Y, Liu K, Xue F, Zhu B, Zhang C (2022). Evolutionary course of the femoral head osteonecrosis: Histopathological - radiologic characteristics and clinical staging systems.. J Orthop Translat..

[8] Konarski W, Pobozy T, Sliwczynski A, Kotela I, Krakowiak J, Hordowicz M (2022). Avascular Necrosis of Femoral Head-Overview and Current State of the Art.. Int J Environ Res Public Health..

[9] Liu J, Li Z, Ding J, Huang B, Piao C (2021). Femoral neck fracture combined with anterior dislocation of the femoral head: injury mechanism and proposed novel classification.. BMC Musculoskelet Disord..

[10] Wang Y, Ma JX, Yin T, Han Z, Cui SS, Liu ZP (2019). Correlation Between Reduction Quality of Femoral Neck Fracture and Femoral Head Necrosis Based on Biomechanics.. Orthop Surg..

[11] Li Z, Shao W, Lv X, Wang B, Han L, Gong S (2023). Advances in experimental models of osteonecrosis of the femoral head.. J Orthop Translat..

[12] Jia Y, Zhang Y, Li S, Li R, Li W, Li T (2023). Identification and assessment of novel dynamic biomarkers for monitoring non-traumatic osteonecrosis of the femoral head staging.. Clin Transl Med..

[13] Zhang W, Du H, Liu Z, Zhou D, Li Q, Liu W (2023). Worldwide research trends on femur head necrosis (2000-2021): a bibliometrics analysis and suggestions for researchers.. Ann Transl Med..

[14] Xu Y, Zeng P (2022). A review and meta-analysis of the survival rate of adult with osteonecrosis of the femoral head treated with transtrochanteric rotational osteotomy.. Med (Baltimore)..

[15] Ma JX, He WW, Zhao J, Kuang MJ, Bai HH, Sun L (2017). Bone Microarchitecture and Biomechanics of the Necrotic Femoral Head.. Sci Rep..

[16] Menger MM, Braun BJ, Herath SC, Kuper MA, Rollmann MF, Histing T (2021). Fractures of the femoral head: a narrative review.. EFORT Open Rev..

[17] Zhang QY, Li ZR, Gao FQ, Sun W (2018). Pericollapse Stage of Osteonecrosis of the Femoral Head: A Last Chance for Joint Preservation.. Chin Med J (Engl)..

[18] Shi W, Zhang X, Xu C, Pang R, Fan Z, Wan X (2022). Identification of Hub Genes and Pathways Associated with Oxidative Stress of Cartilage in Osteonecrosis of Femoral Head Using Bioinformatics Analysis.. Cartilage..

[19] Li G, Liu H, Zhang X, Liu X, Zhang G, Liu Q (2020). The protective effects of microRNA-26a in steroid-induced osteonecrosis of the femoral head by repressing EZH2.. Cell Cycle..

[20] Fu D, Yang S, Lu J, Lian H, Qin K (2021). LncRNA NORAD promotes bone marrow stem cell differentiation and proliferation by targeting miR-26a-5p in steroid-induced osteonecrosis of the femoral head.. Stem Cell Res Ther..

[21] Liu Y, Zong Y, Shan H, Lin Y, Xia W, Wang N (2020). MicroRNA-23b-3p participates in steroid-induced osteonecrosis of the femoral head by suppressing ZNF667 expression.. Steroids..

[22] Wang A, Ren M, Song Y, Wang X, Wang Q, Yang Q (2018). MicroRNA Expression Profiling of Bone Marrow Mesenchymal Stem Cells in Steroid-Induced Osteonecrosis of the Femoral Head Associated with Osteogenesis.. Med Sci Monit..

[23] Wu L, Su C, Yang C, Liu J, Ye Y (2022). TBX3 regulates the transcription of VEGFA to promote osteoblasts proliferation and microvascular regeneration.. PeerJ..

[24] Zhao J, Zhang X, Guan J, Su Y, Jiang J (2022). Identification of key biomarkers in steroid-induced osteonecrosis of the femoral head and their correlation with immune infiltration by bioinformatics analysis.. BMC Musculoskelet Disord..

[25] Huang C, Wen Z, Niu J, Lin S, Wang W (2021). Steroid-Induced Osteonecrosis of the Femoral Head: Novel Insight Into the Roles of Bone Endothelial Cells in Pathogenesis and Treatment.. Front Cell Dev Biol..

[26] Liu J, Han X, Qu L, Du B (2023). Identification of key ferroptosis-related biomarkers in steroid-induced osteonecrosis of the femoral head based on machine learning.. J Orthop Surg Res..

[27] Qi B, Li C, Cai X, Pu L, Guo M, Tang Z (2023). Bioinformatics-Based Analysis of Key Genes in Steroid-Induced Osteonecrosis of the Femoral Head That Are Associated with Copper Metabolism.. Biomedicines..

[28] Xu Y, Jiang Y, Xia C, Wang Y, Zhao Z, Li T (2020). Stem cell therapy for osteonecrosis of femoral head: Opportunities and challenges.. Regen Ther..

[29] Tadesse S, Yu M, Kumarasiri M, Le BT, Wang S (2015). Targeting CDK6 in cancer: State of the art and new insights.. Cell Cycle..

[30] Liu TT, Chen YK,, Adil M, Almehmadi M, Alshabrmi FM, Allahyani M (2023). In Silico Identification of Natural Product-Based Inhibitors Targeting IL-1β/IL-1R Protein-Protein Interface.. Mol..

[31] Vigers GPA, Anderson LJ, Caffe P, Brandhuber BJ (1997). Crystal Structure of the Type-I Interleukin-1 Receptor Complexed with Interleukin-1beta.. Nature ..

[32] Armen RS, Chen J, Brooks C3 (2009). An Evaluation of Explicit Receptor Flexibility in Molecular Docking Using Molecular Dynamics and Torsion Angle Molecular Dynamics.. J Chem Theory Comput..

[33] Zarghi A, Arfaei S (2011). Selective COX-2 Inhibitors: A Review of Their Structure-Activity Relationships.. Iran J Pharm Res..

[34] Samtiya M, Aluko RE, Dhewa T, Moreno-Rojas JM (2021). Potential Health Benefits of Plant Food-Derived Bioactive Components: An Overview.. Foods..

[35] Hewlings SJ, Kalman DS (2017). Curcumin: A Review of Its Effects on Human Health.. Foods..

[36] Nagle DG, Ferreira D, Zhou YD (2006). Epigallocatechin-3-gallate (EGCG): chemical and biomedical perspectives.. Phytochemistry..

