# Supplementary material for: Bioinformatics and Gene Expression Omnibus Analysis of Key Candidate Genes and Pathways Associated with Femoral Head Necrosis
Source: Iran J Pharm Res. 2024 Jun 2;23(1):e145223. doi: 10.5812/ijpr-145223 (PMC11742569; doi:10.5812/ijpr-145223)
Supplement: ijpr-23-1-145223-s001.pdf [file ijpr-23-1-145223-s001.pdf]

Appendix 1. List of the compounds retrieved from the TCMSP database

Compound	PubChem ID	Torsions	Heavy Atoms	MW
3,3'-Diindolylmethane	CID_3071	2	19	246.31
Alliin	CID_87310	5	11	177.22
Aloe emodin	CID_10207	1	20	270.24
Apigenin	CID_5280443	1	20	270.24
Arctigenin	CID_64981	7	27	372.41
Arctiin	CID_100528	10	38	534.55
Astragaloside	CID_5488387	7	55	784.97
Baicalein	CID_5281605	1	20	270.24
Besigomsin	CID_3001662	4	30	416.46
beta-Sitosterol	CID_222284	6	30	414.71
Betulinic acid	CID_64971	2	33	456.70
Bisabolol	CID_10586	4	16	222.37
Campesterol	CID_173183	5	29	400.68
Capsaicin	CID_1548943	9	22	305.41
Chamazulene	CID_10719	1	14	184.28
Cianidanol	CID 9064	1	21	290.27
Cnicin	CID_5281435	6	27	378.42
Curcumin	CID_2889	8	27	368.38
Curcumin	CID_969516	8	27	368.38
Cyanidin	CID_128861	1	21	287.24
Cycloartenol	CID_92110	4	31	426.72
Cycloastragenol	CID_44144539	2	35	490.72
Daidzein	CID_5281708	1	19	254.24
Daphnoretin	CID_5281406	3	26	352.29
Ellagic Acid	CID_5281855	0	22	302.19
Emodin	CID_3220	0	20	270.24
Ephedrine	CID_9294	3	12	165.23
Epigallocatechin gallate	CID_65064	4	33	458.37
Eugenin	CID_10189	1	15	206.20
Eugenitin	CID_3083581	1	16	220.22
Eugenol	CID_3314	3	12	164.20
Ferulic acid	CID_445858	3	14	194.18
Fisetin	CID_5281614	1	21	286.24
Gallic acid	CID_370	1	12	170.12
Genistein	CID_5280961	1	20	270.24
Gingerol	CID_442793	10	21	294.39
Glabridin	CID_124052	1	24	324.37
Glycyrrhetinic acid	CID_10114	1	34	470.68
Hesperidin	CID_10621	7	43	610.56

Isorhamnetin	CID_5281654	2	23	316.26
Kaempferol	CID_5280863	1	21	286.24
Luteolin	CID_5280445	1	21	286.24
Matairesinol	CID_119205	6	26	358.39
Myricetin	CID_5281672	1	23	318.24
Naringenin	CID_439246	1	20	272.25
Naringin 3D	CID_442428	6	41	580.54
Nordihydroguaiaretic acid	CID_4534	5	22	302.37
NSC333050	CID_433563	3	25	348.39
Peonidin	CID_441773	2	22	301.27
Piceatannol	CID_667639	2	18	244.24
Pterostilbene	CID_5281727	4	19	256.30
Quercetin	CID_5280343	1	22	302.24
Resveratrol	CID_445154	2	17	228.24
Rhamnetin	CID_5281691	2	23	316.26
Rivastigmine	CID_77991	6	18	250.34
Scutellarin	CID_185617	4	33	462.36
Sesamin	CID_5204	2	26	354.35
Shogaol	CID_5281794	9	20	276.37
Silibinin	CID_31553	4	35	482.44
Sodium ferulate	CID_5321361	3	14	193.18
Solanine	CID_6537493	8	61	868.06
Sophoricoside	CID_5321398	4	31	432.38
Stigmasterol	CID_5280794	5	30	412.69
Tangeritin	CID_68077	6	27	372.37
Thymol	CID_6989	1	11	150.22
Vanillic acid	CID_8468	2	12	168.15
Vanillin	CID_1183	2	11	152.15
Zerumbone	CID_5470187	0	16	218.34
Zingerone	CID_31211	4	14	194.23

Appendix 2. Molecular interaction analysis of the Top 3 docking hits docked against the target protein linked with FHN

PDB ID	Ligand	Amino acids	Interaction Energy	Interaction Distances
1BI7	Hesperidin	Tyr170	-2.5	2.98 Å
		Met174	-0.05	3.59 Å
		Phe172	-2.5	2.85 Å
		Ser171	-2.25	3.15 Å
		Tyr185	-1.14	3.37 Å
		Glu211	-2.5	2.93 Å
		Thr106	-2.5	3.09 Å
		Leu109	-2.5	2.67 Å
		Arg215	-2.5	2.78 Å
	Naringin	Gln149	-2.5	3.10 Å
		Pro148	-1.6	3.28 Å
		Tyr185	-1.6	3.29 Å
		Tyr106	-2.5	2.78 Å
		Arg168	-2.5	2.91 Å
	Sophoricoside	Arg168	-2.5	2.84 Å
		Gln149	-2.5	2.70 Å
		Tyr185	-2.5	2.65 Å
		Thr106	-0.09	3.58 Å
		Asp110	-2.5	3.06 Å
1HCQ	Hesperidin	Tyr17	-2.31	3.14 Å
		His18	-2.25	2.57 Å
		Tyr19	-2.5	2.77 Å
	Capsaicin	Tyr19	-2.5	3.00 Å
		Gly20	-0.37	3.25 Å
	Naringin	His18	-2.5	2.88 Å
		Tyr19	-2.5	2.74 Å
		Ile35	-2.5	2.93 Å
9ILB	Arctiin	Tyr24	-2.5	2.73 Å
		Leu80	-2.03	3.19 Å
		Gln81	-1.25	3.35 Å
		Leu82	-1.92	2.59 Å
		Leu134	-1.77	3.25 Å
	Naringin	Ser21	-2.5	3.80 Å
		Gly22	-0.92	3.10 Å
		Pro23	-2.05	3.19 Å
		Tyr24	-2.36	2.78 Å
		Glu25	-2.5	2.75 Å
		Lys74	-2.5	2.87 Å
		Leu82	-2.5	2.63 Å
		Val132	-1.76	2.51 Å
	Curcumin	Leu26	-2.5	2.87 Å
		Val132	-1.94	2.53 Å
1DI9	Solanine	Lys152	-1.17	3.36 Å
		Ser154	-2.5	3.04 Å
		Asp168	-2.5	3.03 Å
	Epigallocatechin gallate	His64	-2.5	2.93 Å
		Arg67	-2.04	2.55 Å
		Thr68	-2.5	3.01 Å
		Glu71	-2.5	2.79 Å
		Asp168	-2.5	2.98 Å
		Ala172	-0.05	3.31 Å
		Arg173	-0.78	3.20 Å
		Thr175	-0.47	2.86 Å
		Glu178	-2.5	3.04 Å

	Hesperidin	Ala34	-2.5	2.72 Å
		Gly36	-2.5	3.03 Å
		His64	-2.5	3.10 Å
		Phe169	-2.47	3.11 Å
		Leu171	-1.4	3.13 Å
6BL3	Rhamnetin	His90	-2.5	2.94 Å
		Gln192	-0.68	3.46 Å
		Val349	-0.02	3.60 Å
		Leu352	-2.5	3.01 Å
		Ser353	-2.5	2.90 Å
		Tyr385	-1.94	3.21 Å
		Ser530	-1.68	3.26 Å
	Hesperidin	Lys83	-2.07	3.18 Å
		Tyr115	-2.5	3.08 Å
		Ser119	-0.22	3.55 Å
		Arg120	-2.5	2.86 Å
		Glu524	-2.5	3.08 Å
		Ser530	-2.5	3.10 Å
	Arctiin	Lys83	-2.5	3.05 Å
		Tyr115	-2.16	3.17 Å
3ALQ	Hesperidin	Tyr61	-2.5	2.99 Å
		Ser76	-1.79	3.24 Å
		Ser79	-1.63	2.85 Å
		Gln82	-2.5	2.96 Å
		Asn93	-1.93	3.21 Å
		Lys108	-1.57	3.28 Å
		Arg113	-2.5	3.01 Å
	Curcumin	Arg77	-0.99	3.01 Å
		Lys108	-2.12	2.55 Å
		Cys112	-2.27	3.15 Å
	Epigallocatechin gallate	Ser76	-2.5	2.79 Å
		Arg77	-2.5	3.06 Å
		Ser79	-0.47	3.50 Å
		Gln82	-2.5	2.76 Å
		Lys108	-2.5	2.67 Å
		Cys112	-2.5	3.01 Å
		Arg113	-2.5	2.62 Å
6ZCD (VEGFA)	Curcumin	Ser50	-2.5	3.04 Å
	Shogaol	Glu64	-0.80	3.31 Å
	Resveratrol	Asn62	-0.24	3.55 Å
